# Immediate Filling of Root Canals

**Published:** 1888-12

**Authors:** Wm. H. Potter

**Affiliations:** Boston


### IMMEDIATE FILLING OF ROOT CANALS.

BY WM. H. POTTER, A.B., D.M.D., BOSTON.

READ AT THE OCTOBER MEETING OF THE AMERICAN ACADEMY OF DENTAL SCIENCE, BOSTON.

I have chosen this subject not in order to advocate the immediate filling of all root canals, but in order to discuss the question on both sides, considering the conditions favoring the immediate filling of root canals, and those contra-indicating it. In dealing with this problem, it is necessary that we be guided by correct surgical principles. Though the relation of a tooth to its surrounding tissues is unique, yet the disturbances due to inflammation and abscess must be treated according to the same surgical principle as hold in other parts of the body. I emphasize this point because many seem to work upon the teeth as if they were not vital, and as if they were not subject to the laws governing the rest of the body. The whole question as to whether root canals shall be filled at once or not depends upon the condition of the peridental tissues, and of the root canals. Some of the common conditions requiring root filling are:

1st. An inflamed pulp which must be destroyed and removed. In this condition there has existed, as a rule, no inflammation of the peridental tissues. The removal of the pulp causes a simple wound involving a minute area at the apex of the root. This wound heals quickly by first intention giving rise to no inflammatory products. The conditions are, therefore, favorable for an immediate filling of the root canals. There can be no reason for delay, except in such cases as bleed profusely from the apical artery, thereby rendering the proper drying of the canal impossible.

A second condition requiring root filling is one in which the pulp has died without being exposed to the air, and has given rise to no peridental inflammation. The condition of such pulps commonly escapes notice for a time. When, however, they have been discovered, it becomes a question whether the pulp cavity should be filled at once, or whether a delay is advisable. It is my experience, and I find it also to be the experience of others, that immediate root filling is not wise under the conditions just described. Though the dead pulp may not have given rise to trouble previous to its having been exposed to the air; yet, when so exposed, it seems to furnish particularly good soil for the growth of putrefactive and suppurative germs. If we were able to remove every particle of such a pulp at one sitting, then it would be wise to fill immediately. We are, however, not sure of so doing, and a small remnant being left is capable of being infected with germs and setting up severe peridental disturbance. The influence of germ life should be borne in mind, as it is probable that no suppurative process occurs without the agency of specific germs. The putrefactive process is likewise due to germs. So long as these organisms are kept from a dead pulp, the pulp creates no disturbance, but, when once admitted, they produce characteristic results. The filling of root canals of the class described above should be delayed till the dead pulp has been thoroughly removed and the canals disinfected. We can know that this result has been obtained when the tooth remains comfortable for a short period, after having been tightly filled with cotton and stopped with gutta-percha.

A third condition requiring root-filling is where the pulp, by its death and decomposition, has started up an inflammation or abscess in the peridental tissues. This condition we treat on true surgical principles when we remove the irritant and establish as free drainage as possible for the products of inflammation. Unfortunately, in inflammation about a tooth we cannot get at the seat of the disturbance so readily as we could desire. We must rely on the small apical foramen offering an outlet for the discharge. To close this outlet before all disturbance has subsided and the discharge has ceased seems to me bad surgery and bad dentistry. It may be said, however, that if in such cases the root-canal be stopped at once, subsequent trouble may be met by approaching the abscess through the outer alveolar plate, thus giving exit to the abscess and gaining access to the apical space for treatment. Is it wise, however, to force an inflammatory affection to burrow about in the bony alveolus and effect a passage outwards? Is it, moreover, wise to make an artificial opening through the alveolus? If the abscess forces its own way outwards, it causes the patient intense pain and produces a considerable destruction of bone. The practice of trephining through the alveolus, and so reaching the seat of the abscess, I do not find to be very generally advised in our dental authorities except where distinct fluctuation can be made out. Making an external opening is certainly good surgery if we can be reasonably sure of reaching with the trephine the seat of the abscess, and if we can be sure of not injuring important adjoining tissues.

If one examines the anatomical relation of the dental canal and the tips of the teeth, the intervening space is bound to be very small. In trying to reach this intervening space, which is the usual seat of an abscess, we might very easily enter the dental canal and sever the dental nerve or artery. As the lower jaw, the mental nerve which supplies sensation to the chin leaves the dental canal just between the first and second lower bicuspids at a point often involved in abscess about these teeth. In using a trephine at this point there seems to me considerable danger of severing this nerve. But why this discussion with regard to the external opening of abscesses? Because, if we propose to immediately stop root canals before the peridental tissue has become normal we must be prepared to let out the discharge some other way, or else require our patients to endure a considerable amount of pain while the abscess finds its own way out.

I have heard it said of one who practised the immediate filling of root canals that he was very skillful in the use of the alvelor trephine. It seems to me better as a rule to cure the inflammation through the apical foramen rather than to force or make an exit through the alveolus.

A fourth condition to be described is that in which an artificial opening or fistula has already been established as the result of a dead pulp and consequent inflammation. Such cases having an exit for products of inflammation independent of the root canals are much easier of treatment than the foregoing. As soon as the root canals can be made clean, they should be filled.

When root canals are to be filled at one sitting the best method seems to me to be as follows: Make free access to the pulp cavity, enlarge not only the entrance to the root canal, but its entire length. For enlarging root canals, drills which cut ahead are unsafe.

Reamers like the Morey drills which follow the canal and cut at the sides are the safest and most useful. Use a large size drill first, then smaller ones in succession. Even these drills should not be forced, but should be gently advanced only so far as they go easily. There are two reasons for enlarging root canals.

1st. It is easier to fill enlarged canals than those of natural size.

2d. The mechanical removal of the dentine adjacent to the pulp is the best way of disinfecting that tissue. It takes time to disinfect root canals by drugs, and we cannot always be sure that the drugs which we apply do their work. If, however, the infected substance is to a large extent removed bodily the health of the remaining dentine is greatly promoted. I am not an advocate of the extensive drilling out of root canals as practised by some. A process which often results in the perforation of the root and leaves broken instruments to give rise to serious trouble. I do, however, believe in the careful enlarging of root canals to remove decomposable or decomposing substances. When root canals are not enlarged, broaches are commonly used in many ways and not without risk. The peridental tissues may be by them infected through uncleanliness of the instrument or by forcing up putrifying material.

If root canals are to be filled immediately, great care must be given to small details; an antiseptic condition must as far as possible be established and maintained.

While the enlargement of root canals should be the most important antiseptic measure, yet there may be parts of the root canal not completely reached by this process. Certain drugs are therefore of value. First to be mentioned is peroxide of hydrogen, which has the power of converting decomposing substances into harmless ones. Not being a violent caustic, it does not close the tubuli to the entrance of its medicinal power. Having the property of disingaging bubbles of gas as long as any decomposing substance is left, it affords a correct guide for the duration of its application.

Some time ago I had occasion to extract a tooth which had become thoroughly infected by decomposition; it was really a dead tooth. This I immersed in peroxide of hydrogen for several hours, at the end of which time a complete renovation had taken place. The tooth, though kept for some time afterwards, never had the slightest odor. I think no other disinfectant would have done as well. Peroxide of hydrogen should be kept well stoppered in a cold place, the colder the better. Cold condenses the gas and it is less likely to escape. When needed for use a small bottle should be filled. Even with care this drug deteriorates very rapidly; it is especially difficult to keep in the summer time. Other drugs are of value, such as alcohol, which is a detergent and dryer; carbolic acid 5 per cent. and corrosive sub. 1–1000, both of which latter are powerful germicides.

